# Oxygen Vacancy Dynamics in Different Switching Modes of Hf_0.5_Zr_0.5_O_2−δ_

**DOI:** 10.1021/acsnano.5c07038

**Published:** 2025-08-06

**Authors:** Judith Knabe, Kalle Goss, Yen-Po Liu, Evangelos Golias, Alexei Zakharov, Iulia Cojocariu, Matteo Jugovac, Andrea Locatelli, Tevfik O. Menteş, Denis Céolin, Alexander Gutsche, Daisy Gogoi, Moritz L. Weber, Rainer Timm, Regina Dittmann

**Affiliations:** † 163337 Peter Grünberg Institut (PGI-7), Forschungszentrum Jülich GmbH, 52428 Jülich, Germany; ‡ MAX IV Laboratory, 5193 Lund University, 22100 Lund, Sweden; § 18474 Elettra-Sincrotrone Trieste S.C.p.A, S.S 14-km 163.5 in AREA Science Park, Basovizza, 34149 Trieste, Italy; ∥ Synchrotron SOLEIL, l’Orme des Merisiers, Saint-Aubin, F-91192 Gif-sur-Yvette Cedex, France; ⊥ Peter Grünberg Institut (PGI-6), Forschungszentrum Jülich GmbH, 52428 Jülich, Germany; # NanoLund and Department of Physics, Lund University, Lund 22100, Sweden

**Keywords:** HZO, resistive switching, ferroelectric switching, HAXPES, XPEEM, spectroscopy

## Abstract

HfO_2_, one of the most common materials in resistive switching devices, can stabilize in a ferroelectric orthorhombic phase, enabling two nonvolatile polarization states via oxygen displacement in the unit cell. Under certain conditions, ferroelectric and resistive switching can coexist, independently addressable, within one device. This study employs *operando* spectroscopic analysis to elucidate the role of oxygen in both switching processes. A conductive filament is identified through a local valence change at the oxide surface via X-ray Photoelectron Emission Microscopy, allowing vacancy density and filament diameter evaluation. This provides well-founded experimental evidence of a conductive filament in orthorhombic Hf_0.5_Zr_0.5_O_2−δ_ (HZO) in application-relevant device geometry. Depth-dependent changes in the electronic signature of HZO and La_0.8_Sr_0.2_MnO_3−δ_ (LSMO) with ferroelectric field cycling are identified by Hard X-ray Photoelectron Spectroscopy. Polarization-dependent shifts in the Hf core level align with the oxygen vacancy migration during ferroelectric switching. Fatigue-related vacancy generation causes an inhomogeneous reduction that does not propagate into the bottom electrode and extended domain pinning at the HZO/LSMO interface. This highlights the importance of interface engineering for the ferroelectric performance and of the oxygen affinity of the bottom electrode for both switching regimes.

## Graphical abstract

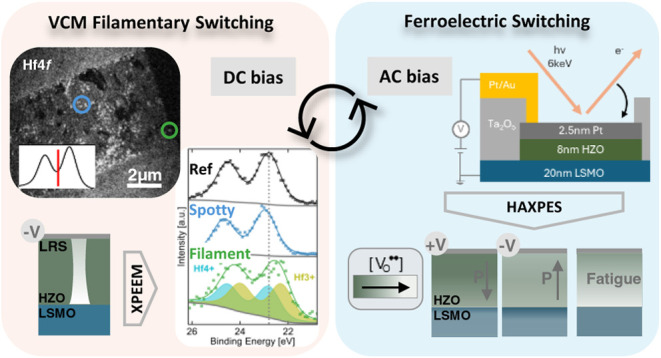

Hafnium dioxide can be stabilized in a metastable ferroelectric orthorhombic phase, where the displacement of oxygen atoms in the unit cell gives rise to two nonvolatile polarization states.^1^ Besides that, HfO_2_ is commonly used as an oxide layer in resistive switching devices, where a filament of oxygen vacancies modulates the device resistance upon an applied bias. Conventionally, the formation of such a filament involves a soft breakdown of the oxide from the pristine state into the low-resistive state (LRS). Subsequently, a partial rupture and regeneration of the filament changes the Schottky barrier at one oxide/electrode interface, enabling different stable resistance states.^2,3^ The irreversible character of the initial filament formation prevents subsequent access to the ferroelectric switching.^4^ There are few reports on both switching modes coexisting, mostly with limited reversibility back to ferroelectric switching and generally involving electroforming to initiate the switching operation.^5–7^ It was shown that under specific conditions in epitaxial Hf_0.5_Zr_0.5_O_2−δ_ (HZO), neither an initial electroforming nor a deep reset is required to switch between a high resistance state that resembles the pristine resistance and a low resistance state. Following this, ferroelectric switching was observed coexisting with filamentary resistive switching in the same device using a La_0.8_Sr_0.2_MnO_3−δ_ (LSMO) bottom electrode. Both nonvolatile switching mechanisms involve oxygen and can still be addressed independently and interchangeably.^8^ What distinguishes them is the different biasing time scale and the expected spatial spread of connected electrochemical changes.

The filamentary nature of other switching oxides used in valence change memory (VCM) based applications, such as SrTiO_3_, TiO_2_, and Ta_2_O_5_, has already been explored.^9–12^ In contrast, experimental investigations on HfO_2_ remain limited, particularly lacking studies on systems with oxide-based electrodes and realistic biasing schemes. Consequently, further experimental evidence on the oxygen vacancy concentration in the filament and regarding different material systems is of great interest.

In this work, we extend experimental observations to an epitaxial model-system with an oxide electrode that serves as an oxygen reservoir and internal current limitation. This is especially interesting due to the forming-free switching behavior and exclusion of an external current compliance. A higher oxygen diffusion in crystalline HZO and the phase-dependency of resistive switching characteristics,^13,14^ can cause deviations from findings on amorphous and metal/oxide systems. Further, this work contributes to understanding a possible breakdown mechanism of ferroelectrically switching hafnia-based systems.^15–17^

The heterostructures of this work consist of epitaxial Hf_0.5_Zr_0.5_O_2−δ_ on La_0.8_Sr_0.2_MnO_3−δ_ buffered SrTiO_3_. This material system has been shown to exhibit stable ferroelectricity in a wide growth window due to the specific lattice mismatches and an interface interaction between the LSMO bottom electrode and the HZO switching layer.^18–21^ Oxygen vacancies and their (re)­distribution have also been shown to critically impact the ferroelectric characteristics of hafnium oxide-based systems.^22–24^ Structural changes related to oxygen movement with applied bias have been demonstrated in HZO and across HZO/LSMO interfaces.^25^ Nevertheless, depth-dependent chemical changes in HZO and LSMO upon field cycling, as well as the related degradation mechanism, remain a point of discussion and ongoing research. Several effects can contribute to or superimpose the initial oxygen vacancy migration during ferroelectric switching, a selection of which includes the internal field generated by polarization charges, which can cause vacancy (re)­migration on longer time scales, or surface adsorbates contributing surface charges on exposed oxide surfaces.^26,27^ To study the oxygen vacancy redistribution in HZO and LSMO as closely to the ferroelectric switching as possible, with representative electrical treatment and device geometry, an *operando* experiment design is crucial. It adds further significant benefits by eliminating the impact of device-to-device variability, which otherwise complicates the comparison of different devices in different states.

Polarization charges are expected to impact the top and bottom interface of the device, depending on the polarization direction. Therefore, high-energy X-rays are necessary to probe the surface as well as the buried HZO/LSMO interface. With *operando* HAXPES, analyzing the line shape and position of core-level spectra reveals correlated chemical and electronic changes at the oxide surface and the buried HZO/LSMO interface.

This technique has already been successfully utilized to quantify the oxygen vacancy concentration in Si:HfO_2_ due to scavenging by a Ti layer with in situ applied field cycling.^28^ HAXPES was also used to determine an interface reduction of epitaxial HZO induced by capping.^29^

We identify depth-dependent changes in oxygen vacancy concentration and distribution in the HZO upon field cycling. The observed inhomogeneous reduction is associated with an overall increase in oxygen vacancy concentration and their preferential accumulation near the underlying LSMO. Polarization direction-dependent core-level shifts are identified and assigned to the respective oxygen migration. Mn spectra of the underlying LSMO bottom electrode confirm the migration between the two polarization states. Changes in the bottom electrode are insignificant, despite increased vacancy density in the fatigued HZO.

In the resistive switching mode of HZO, we aim to identify the highly localized filaments that drive resistance changes. Achieving this requires high spatial resolution while also mitigating reoxidation of the filaments, presenting a significant challenge. We approach this by switching a device into the LRS in situ via a graphene top electrode. Filament regions are identified by a local valence change at the electrode/oxide interface via surface-sensitive X-ray Photoelectron Emission Microscopy (XPEEM) through the graphene top electrode. This experimental technique and device layout have already been shown to be a suitable tool for investigating memristors, such as Ta_2_O_5_ and SrTiO_3_ devices.^12,30^ The oxide core-level spectra exhibit a significant suboxide contribution, and an upper limit of the filament size can be estimated, demonstrating the experimental characterization of a previously unexamined filament in orthorhombic hafnium (zirconium) oxide.

## Results

### Resistive Switching

The highly localized filaments responsible for the resistance change require spatially resolved spectroscopy. To investigate the electrochemical structure of the filaments in HZO, the material system of STO/LSMO/HZO, common to all investigations in this work, is covered with graphene as a top electrode. In this system, the active interface of the filamentary switching mechanism, i.e., where the resistance modulation takes place, is positioned at the top. This allows surface-sensitive XPEEM measurements to effectively detect related changes. The interchangeable ferroelectric and filamentary-type resistive switching, as previously demonstrated for this material system,^8^ is confirmed in Figure 1 for identically fabricated LSMO/HZO films, utilizing standard capacitor structures with Pt top electrodes. Further characterization of this dual-mode operation is demonstrated elsewhere.^8^

**Figure 1:**
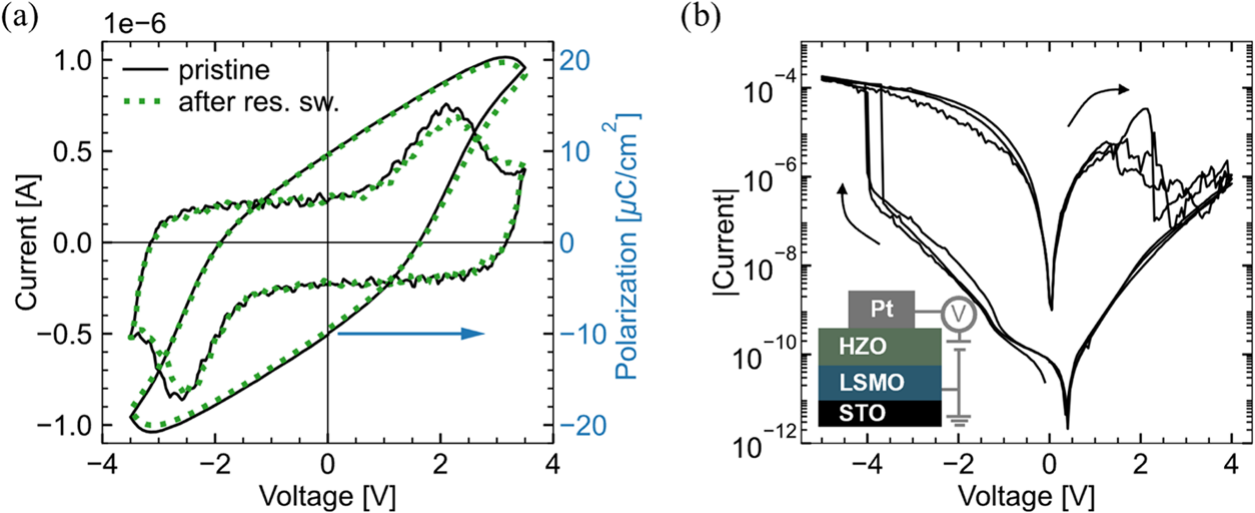
Ferroelectric and resistive switching of LSMO/HZO/Pt measured in the same device. (a) Ferroelectric *I*–*V* and *P*–*V* loops measured at 1 kHz in the pristine state and after one set of resistive switching. (b) Quasi-static *I*–*V* curves of three consecutive cycles of resistive switching, applied after the pristine measurement in (a). Pt top electrodes consist of squares with 20 μm edge length.

Graphene, as a photoelectron-transparent top electrode, improves the signal yield from the underlying oxide layer compared to Pt or Au, which is conventionally used in memristive devices.^30^ Figure 2a shows a scanning electron microscope (SEM) image of the device geometry in the top view. The device is formed at the contact area between the graphene and the HZO on LSMO. Gold contacts on the side ensure a reliable electric contact between the graphene and the sample holder for in situ biasing. LSMO serves as the bottom electrode and is contacted via wire bonding. The device was switched into the LRS to establish a filament for the spectroscopic investigation of the oxide surface. The corresponding switching curve and comparable *ex-situ* switching can be found in Figure S2. The Hf 4*f* measurement in Figure 2b,c of a device in the LRS shows several interesting features. The images were extracted at two different binding energies to highlight deviations from the generally stoichiometric HZO surface. This way, the contrast features in the images can give an indication of electrochemical changes directly correlated to the binding energy chosen for the image. On the higher binding energy side of the Hf 4*f*_7/2_ peak, a bright, spotty structure appears that is inhomogeneously distributed over the device area. The zoom-in in Figure 2c reveals a contrast feature at the bottom right corner of the device that stands out at lower binding energies. The roughly elliptical spot can be estimated at 160 × 100 nm^2^ in diameter, which serves as an upper estimate of the actual filament diameter, given the graphene layer on top of the HZO and a microscope resolution minimum of about 20 nm. The asymmetry of the filament observed in the experiment could result from an adjacent subfilament.^31,32^ Direct comparison of the Hf 4*f* spectra in Figure 3a reveals that the spotty structures are unchanged in shape, with an about 0.2 eV shift to higher binding energies. We understand this rigid shift in the spotty structure as a relatively weaker reduction in the HZO layer. In this context, oxygen vacancies serve as *n*-type dopants, effectively shifting the Fermi level; therefore, raising the binding energy of core-level electrons, as has been observed before, for example in Ta_2_O_5–*x*_.^12,33^ Figure S5 in the Supporting Information shows two equivalent devices in the pristine state that do not show such spotty structures with shifted binding energy, emphasizing their emergence through resistive switching.

**Figure 2:**
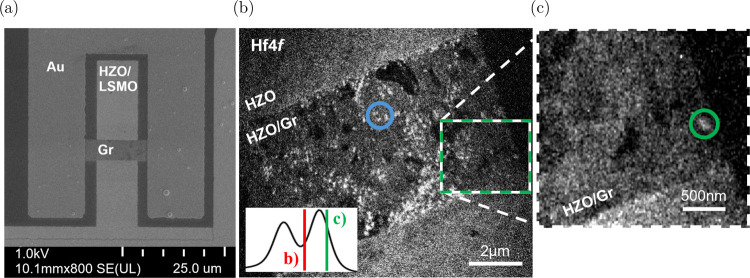
(a) Scanning electron microscopy (SEM) image of the fabricated device structure. The device comprises an STO/LSMO/HZO stack with a graphene (Gr) top electrode. Gold (Au) electrodes are visible on either side, providing electrical contacts to the sample holder. (b) XPEEM Hf 4*f* image of a device in the LRS acquired at 200 eV photon energy. Image extracted at 23.7 eV binding energy. The inset in the bottom left shows the relative position of the extracted images in (b and c). The blue circle highlights the area from which the following spectra are extracted. (c) Zoom-in on the filament region in (b). Image extracted at 22.5 eV binding energy. The green circle highlights the filament.

**Figure 3:**
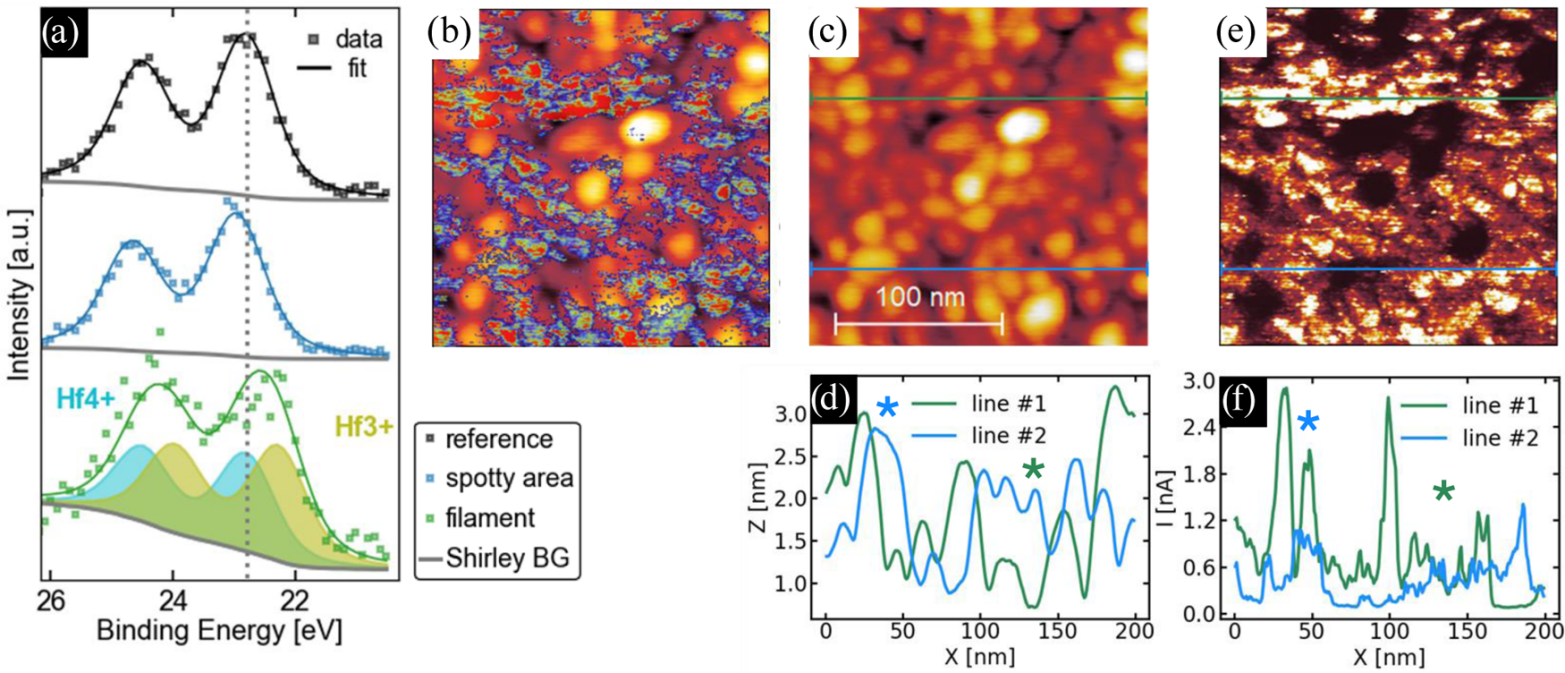
(a) XPEEM Hf 4*f* raw data (squares) and envelope of fit components (line) extracted from the different regions in Figure 2b, c in comparison with a reference region obtained in the vicinity. The spectra are fitted with a Shirley background and Voigt doublets. The filament spectrum is shifted and widened, justifying a suboxide doublet. Conductive AFM measurement and XPEEM Hf4*f* spectra. (b) Overlay of the topography of the HZO surface in a 200 nm by 200 nm region in (c) and the current map acquired simultaneously with the topography with a sample bias of +7 V, electrically connected to the bottom electrode in (e). (d) Scan profiles along the green and blue lines shown in (c). (f) Current profiles of the green and blue lines shown in (e).

The spectrum of the filament spot is significantly broadened and shifted to lower binding energies, corresponding to an additional Hf^3+^ doublet that makes up 54% of the total Hf 4*f* peak. No suboxide is present in the reference spectrum. Details regarding the fit can be found in the Experimental Section. This suboxide contribution corresponds to an oxygen vacancy concentration of 13.5%, assuming charge neutrality, which equals an oxygen vacancy density of 75·10^26^m^–3^ that can be assumed at the HZO top interface. For details on the calculation, see Eq S3 in the Supporting Information. Due to the low signal-to-noise ratio in the filament spectrum, the deduced vacancy density is subject to a significant error.

Additionally, the Supporting Information presents data from a second filament in a non-*operando* device with a more general geometry and in situ delaminated Au top electrode (Figure S4a). These data support the observed range of filament diameter (100 nm) and vacancy concentration (14.5%, Figure S4b), further validating the universality of the chosen *operando* device geometry.

Conductive atomic force microscopy (C-AFM) measurements of the HZO surface provide further information about the origin of the spotty regions identified in Figure 2b. Figure 3e,3f reveal spots and clusters of elevated conductivity in the range of 30 to 100 nm. The overlay of topography and current map in Figure 3b and a direct comparison of the two exemplarily extracted profiles in blue and green (Figures 2d and 3f) demonstrate that an elevated conductivity is partly, but not solely related to a locally reduced thickness. This can be exemplarily understood from the location of the blue and green asterisks along the profiles in Figure 3d,3f. In blue, a location is marked that exhibits increased current at a peak in topography, while the green asterisk marks a valley with no significant change in current, both demonstrating a discrepancy between topography and current.

### Ferroelectric Switching

Angle-dependent Hard X-ray Photoelectron Spectroscopy (HAXPES) measurements of the Hf 3*d* core level were conducted to investigate ferroelectric switching. Devices consist of the same STO/LSMO/HZO material system described before. Here, they are modified with an ultrathin Pt top electrode instead of graphene, since HAXPES allows for a larger escape depth of photoelectrons. The devices are surrounded by an insulating Ta_2_O_5_ layer and an Au side-contact enabling in situ biasing, Figure 4a. Figure 4b presents an SEM image of a device. Corresponding in situ and ex-situ current responses, following the PUND scheme, are provided in Figure S7. The elongated device geometry was chosen to maximize the device area probed by the incident X-ray beam. Ferroelectric *I*–*V* and *P*–*V* loops of equivalent devices with a similarly thin noble metal electrode (here Rh) are shown in Figure 4c,4d, demonstrating unambiguous ferroelectric switching. Smaller device areas were employed here for an improved characterization of the ferroelectric properties of the LSMO/HZO films. Details on the fabrication can be found in the Experimental Section. Measurements were performed on the same device in its pristine state, in both polarization orientations, and in a fatigued state following <10^6^ switching cycles. This *operando* approach ensures highly comparable spectra by eliminating device-to-device variability. Further, due to the immediate measurement after in situ biasing, vacancy drift due to the internal field caused by the polarization can be neglected. For each state, spectra were collected at three distinct takeoff angles to capture qualitative depth-dependent variations. The Pt 4*f*_7/2_ peak from the platinum top electrode provides a reference, allowing alignment of the binding energy of all spectra. A Voigt peak profile was used to model the Hf 3*d*_5/2_ peak, while a Donjach–Sunjich peak shape was applied to the Pt reference peak. Details on the fitting can be found in the Experimental Section.

**Figure 4:**
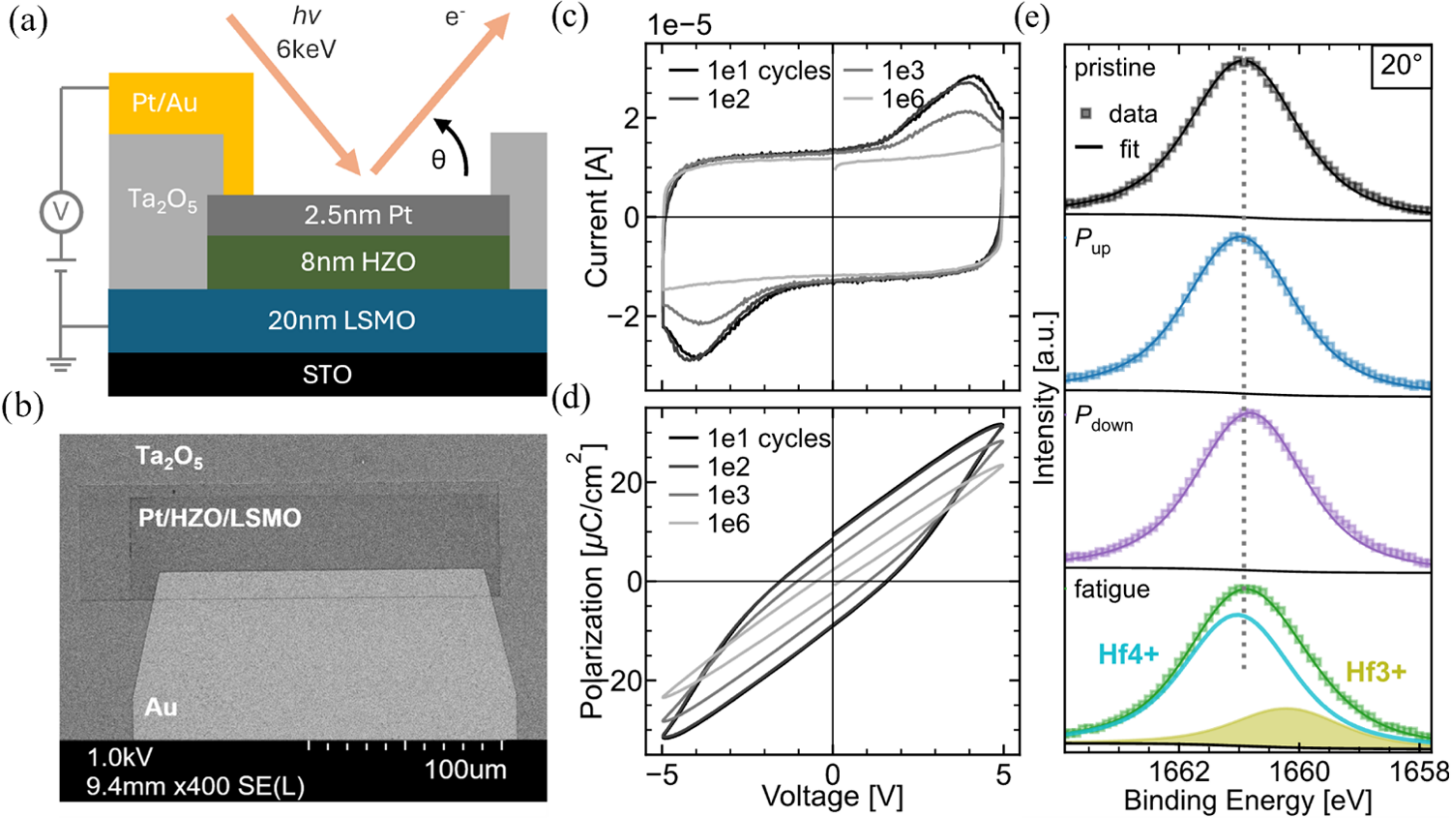
(a) Schematic HAXPES device cross-section. The black arrow indicates the takeoff angle Θ. (b) Device top-view SEM image. (c) *I*–*V* and (d) *P*–*V* loops of equivalent LSMO/HZO layers measured for continuous field cycling in smaller (2500 μm^2^) devices with ultrathin Rh top electrode. Measured and cycled at 5 kHz. (e) HAXPES Hf 3*d*_5/2_ core-level spectra: Raw data (squares) and envelope of fits (line) of the Hf 3*d*_5/2_ peak for the different states at Θ = 20°. The dashed line marks the peak maximum of the pristine state.

Figure 4c presents a comparison of the Hf 3*d*_5/2_ peak position for different device states, exemplarily for the most surface-sensitive measurements. These data reveal subtle peak shifts that vary with the device’s state. Detailed peak fitting for each state confirms that the broadened peak of the fatigue state indicates a minor suboxide component. The other states show no suboxide contribution. To analyze the spatial distribution of the binding energies, we summarized the positions of all fitted peaks in Figure 5. For the fatigue state, the Hf^4+^ component is used. Additionally, measurements were conducted on a second sample (referred to as device #2 in Figure 5, excluding the fatigue state), to verify the reproducibility of subtle peak shifts. A trend shows that the binding energy increases toward the bottom electrode (i.e., from Θ = 45 to 60°) indicated by the gray arrow. In the pristine state, however, the 60° measurement clearly deviates from this trend. This decrease in binding energy toward the bottom, in the pristine state, is attributed to an oxygen-rich condition in the HZO layer at the interface with the LSMO bottom electrode. Such oxidation is consistent with the difference in free energy gain between these materials and has been previously documented for this material system.^8,65^ Considering only up to a few monolayers at the interface, signs of reduced LSMO, but also oxidized LSMO and reduced HZO have been found.^21,34–36^ Such differences might arise from varying deposition conditions, stoichiometry and the extent of the investigated interface region.

**Figure 5:**
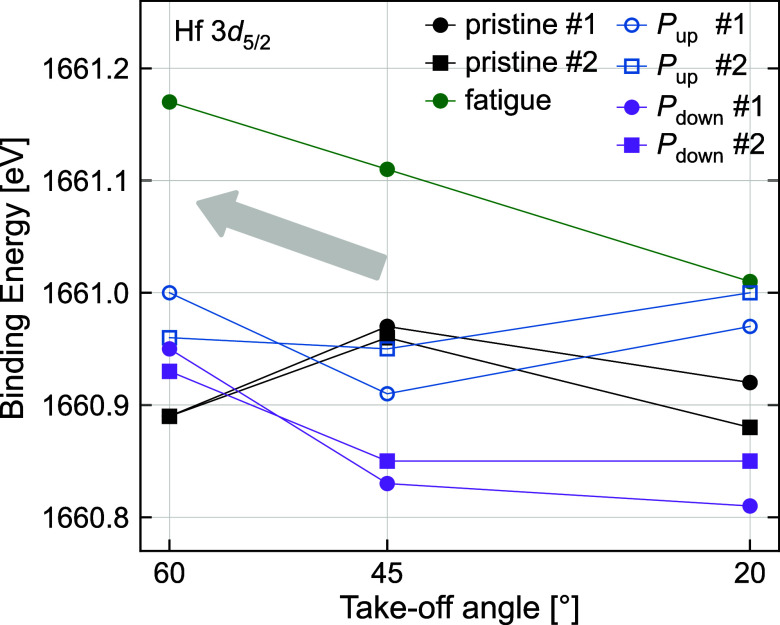
Binding energy positions of the fitted Hf 3*d*_5/2_ peak for two identical devices (device #1 and device #2) measured in the pristine, *P*_up_, *P*_down_, and fatigued state at three takeoff angles. The Θ = 60° angle has the highest average information depth, while 20° has the lowest information depth.

After the pristine state, field cycling induces a binding energy gradient, which becomes more pronounced with increasing cycles (see fatigued state in green in Figure 5). A direct comparison of the two polarization directions shows a clear difference in binding energy of about 0.15 eV at the most surface-sensitive measurement (20°), which diminishes toward the bottom interface. The results consistently demonstrated the observed trends, affirming that the binding energy shifts, despite being small, can be reliably reproduced by the same electrical treatment. In the fatigued state, the binding energy is shifted most to higher values and relatively similar suboxide contributions (between 17 and 25%) appear at all takeoff angles (see Figure 4c for 20° and Figure S6a,b for 45 and 60°). The shifts of the Hf 3*d*_5/2_ binding energy are complemented by the Mn 2*p* spectra obtained from the underlying LSMO bottom electrode. Here, only the measurements at 45 and 60° (Figure 6a,b, respectively) provide a sufficient signal-to-noise ratio, which is consistent with a high surface sensitivity at smaller takeoff angles. Minor changes of the peak maximum compared to the pristine state, indicated by the dashed line, provide information on the Mn^4+^ to Mn^3+^ ratio and, therefore, on valence changes of the LSMO. Rigid shifts, as in the Hf core-level, are not considered for Mn, since differences in the peak shape are visible between different states. Difference spectra of the Mn 2*p* measurements in Figure 6a,b with respect to the pristine state can be found in Figure S8.

**Figure 6:**
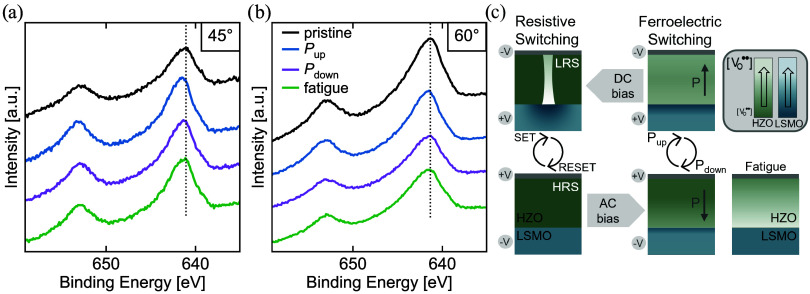
HAXPES Mn 2*p* spectra measured in the four different states, at (a) Θ = 45° and (b) 60° (highest information depth). As a guide to the eye, the dashed line indicates the peak maximum of the pristine state in the respective graph. (c) Schematic overview of the two switching modes in LSMO/HZO and findings regarding the respective oxygen vacancy (re)­distribution in different states. A lighter color indicates a higher oxygen vacancy concentration.

A shift of the maximum toward higher binding energies reflects a relative increase of the Mn^4+^ fraction, while vice versa, a shift to smaller energies reflects an increase in Mn^3+^.^37^ At lower average information depth (45°), the peak maximum of the *P*_up_ state clearly lies at higher binding energies, while the *P*_down_ state might show a minute change in the same direction. The fatigue state is approximately equal to the pristine state. At larger information depth (60°), the *P*_up_ state shows a smaller change to higher binding energies than at 45°, and the other states remain approximately unchanged compared to the pristine state.

## Discussion

### Resistive Switching

The filament diameter of approximately 100 nm (Figures 2c and S4a) and the estimated vacancy concentration of about 14% (average of Figures 3a and S4b) lie in the same order of magnitude as findings on amorphous Ta_2_O_5_ (120 nm and 20%) in a similar experiment design.^12^ There are other, albeit limited, experimental findings regarding filaments in HfO_2_ obtained under various experimental conditions. Filament diameters of 20–100 nm have been found in polycrystalline HfO_2_/TiN using C-AFM and transmission electron microscopy (TEM) based techniques.^38,39^ Oxygen deficiency was identified by Calka et al. estimating up to 55% deficiency.^38^ These findings provide an initial basis for understanding, yet lack full comparability with typical switching behavior in devices, as switching was induced using a C-AFM tip rather than standard switching protocols.

Regarding amorphous HfO_2_, sub-10 nm filaments have been observed in Hf/a-HfO_2_ with C-AFM tomography after top electrode removal. The devices were embedded in a 1T1R cell, exhibiting low switching voltages of < ± 1 V and a fast current compliance of 50 μA due to the transistor in series.^40^ In addition, metallic filaments of 5–15 nm in diameter have been observed in a-HfO_2_ in TEM images.^41,42^ A significantly smaller reduction of the switching oxide has been found in a-ZrOx/Ta via XPEEM, though likely underestimated due to a limited spatial resolution.^43^ Deshmukh et al. utilized Scanning Thermal Microscopy (SThM) to investigate a-HfO_2_ on TiN with either TiN or graphene (single and double-layer) as the top electrode. The findings provided only indirect evidence of filament size by measuring hot spots of >120 nm under steady-state bias, which simulations suggested correspond to filament diameters of less than 20 nm. Nevertheless, the study found a minimal difference in hot spot diameters between TiN and graphene as top electrodes, further supporting the suitability of graphene as a representative top electrode.^44^

Further, from a simulation point of view, vacancy concentration and size of the filament in HfO_2_ and ZrO_2_ have been assigned in several studies.^45–47^ These findings show a significant discrepancy with the pronounced reduction in the experimental studies mentioned above, some of which exhibit even near-metallic states.

Due to the different experimental designs, material systems, and electrical treatments, a direct comparison with the studies mentioned is of limited significance. Nevertheless, an assessment of the results in relation to simulation studies is given below. The found filament diameter aligns with simulation results on Pt/HfO_2_/TiOx/Ti/Pt (90 nm) and Pt/ZrOx/Ta/Pt (50–70 nm), hinting toward a general consistency between experimental and theoretical findings.^45–47^ The oxygen deficiency estimated from the deconvolution of the Hf core-level can be translated into an oxygen vacancy concentration of approximately 78·10^26^ m^–3^ at the surface as an average of Figures 3a and S4b. This is in the same order of magnitude of literature values obtained from the same studies (20·10^26^ m^–3^ for HfO_2_/TiOx and 140–160·10^26^ m^–3^ for ZrOx/Ta). The slightly higher concentration than in the HfO_2_/TiOx system could be caused by differences in the current compliance and the higher switching voltages (Figure S2). The ZrOx/Ta system likely exhibits a higher vacancy density due to the probable oxidation of the Ta layer at the interface. Ultimately, device-to-device variability and cycle-to-cycle variability of filamentary switching are influenced by alterations in filament configurations.^31^ Consequently, it is not feasible to establish a definitive, final filament size and composition.

The observed rigid shift in the spotty structure in Figure 3a resembles a weaker reduction in the HZO layer, compared to the filament. This spectral shift highlights the influence of oxygen vacancy-induced doping on the electronic structure, emphasizing the role of vacancies in locally modulating the material’s electronic properties outside of the filament. Their origin might lie in local structural defects that have a lowered oxygen vacancy formation energy, such as o-/o-phase grain boundaries or residual paraelectric m-/o-phase boundaries,^48–50^ although no significant monoclinic phase fraction was found in XRD measurements (Figure S1a). This might possibly be explained by a limited coherence length of nanosized phase fractions. Further, the HZO surface does not show any topographical features on a micrometer scale (Figure S1b). The conductivity variations in Figure 3e, found in pristine HZO via C-AFM, support inherent structural variations as an origin of preferential oxygen vacancy paths. The size of elevated conductivity regions is in the order of the found filament and of the substructures in the spotty regions (cf. Figure 2b).

Furthermore, the reported grain and domain sizes of HZO are approximately in the range of the conductivity spots and clusters in Figure 3e.^51–53^ From this, it can be assumed that the filament responsible for resistive switching originated from one of many preferential paths. Accelerated by Joule heating,^54^ a substantial reduction occurs during switching into the LRS, which distinguishes the switching filament from other preferential regions that remain comparably weakly reduced. The abundance of potential preferential paths highlights the inherent cycle-to-cycle variability of filamentary-type resistive switching.^31,32^

### Ferroelectric Switching

The observed peak shifts are attributed to the influence of the applied voltage, which sets the device into distinct polarization states: a positive voltage applied to the Pt top electrode induces the *P*_down_ state, whereas a negative voltage establishes the *P*_up_ state. This applied voltage either repels or attracts charged oxygen vacancies, leading to corresponding variations in the *n*-type doping level within the HZO. These changes in doping concentration result in binding energy shifts that correlate with the device’s polarization and fatigue state.

Given that fatigue is commonly linked to oxygen vacancy formation, the inhomogeneous reduction through the HZO thickness likely arises through an overall increase in vacancies and their preferential pinning at the underlying LSMO, which might further be connected to a dead-layer emerging at the bottom interface.^22,55,56^ It should be noted that the vacuum environment could possibly promote oxygen vacancy formation in comparison to ambient switching conditions. Nevertheless, this can be assumed negligible since no signs of reduction could be identified after continuous exposure to the hard X-ray beam, see Figure S9 in the Supporting Information. Depending on the polarization direction, a shift of the HZO core levels can be found, resembling the respective oxygen migration. The polarization-dependent change is most pronounced at the surface, which can be explained by a compensating impact of oxygen migration from and to the LSMO bottom electrode and/or the superimposed vacancy gradient/pinned layer near the LSMO interface. The higher binding energies of the Hf 3*d*_5/2_ peak in the *P*_up_ state, compared to the *P*_down_ state in Figure 5, indicate an increase in *n*-type doping via additional vacancies, reasoned by vacancies migrating into the HZO due to the applied negative voltage. This is supported by the Mn spectra, showing a change to an increased relative Mn^4+^ fraction (located at higher binding energies than Mn^3+^) in the *P*_up_ state, resembling an oxidation of the LSMO as the counterpart to the reduction in the HZO. This effect is more pronounced in the Θ = 45° spectrum compared to the more bulk-sensitive 60° spectrum, emphasizing that the HZO-induced chemical modifications in the LSMO are primarily confined to the interface region, with minimal impact on the bulk. The *P*_down_ state does not induce a clear spectroscopical change in the LSMO. A minute shift to a higher Mn^4+^ fraction might be present for Θ = 45° only. Despite the observed increase in oxygen vacancies with field cycling and the formation of a suboxide phase in the Hf layer, the Mn 2*p* spectra reveal no substantial increase in Mn^3+^ in the fatigue state. Instead, a minimal shift to a higher Mn^4+^ fraction might be present for Θ = 45°, similar to the *P*_down_ state. This allows the assumption of a state of saturated reduction in the LSMO, already in the pristine state, from which the material does not deviate significantly, despite a positive voltage applied (*P*_down_) or extensive field cycling (fatigue state). This stability in the Mn core level implies a limited influence of the vacancies in HZO on the LSMO, corroborating the interpretation that chemical interactions are mainly restricted to the interfacial region, in line with a study that found some oxygen-deficient reordering in La_0.67_Sr_0.33_MnO_3_ close to the interface with rhombohedral HZO at 1 kHz, in the same regime as the ferroelectric switching applied here. In contrast to that, they found the oxygen-deficient Brownmillerite phase in the whole LSMO layer upon positive DC bias over an extended time.^25^

Our observations suggest that, although oxygen exchange occurs between the HZO and LSMO, a majority of the generated oxygen vacancies remain localized within the HZO. These vacancies appear to be immobilized, accumulating near the bottom interface of the HZO layer, as seen in the gradient in the Hf 3*d* binding energy, Figure 5.

This finding suggests that the ferroelectric degradation in this system is mainly dominated by domain pinning, which can be related to charge trapping by the oxygen vacancies,^22,49^ rather than an increase of leakage current and a dielectric breakdown.^55,57^ It has been shown that higher applied fields can recover such degradation by oxygen vacancy redistribution and detrapping,^58^ but it can be assumed that higher fields only detrap temporarily, given that an inhomogeneous vacancy distribution seems intrinsic to the material system. Further, an initial monoclinic phase fraction has been shown to reduce domain pinning, although at the cost of an inferior ferroelectric performance.^59^

Our findings generally align with those of Hamouda et al., who detected a vacancy increase at the surface upon field cycling in TiN/HZO/TiN via XPEEM measurements.^26^ A comparison of the polarization state dependence on the vacancy concentration cannot be drawn, due to the very different impact of the internal field on their longer (non-*operando*) time scales. Deviating results have been found for HZO with IrO_2_ electrodes, where an oxygen-rich interface emerged.^60^ This difference could be attributed to the expectantly different interface between the epitaxially grown LSMO/HZO and the polycrystalline HZO with sputtered IrO_2_ and further the significantly less favorable oxidation of Ir in comparison with Mn.

Recently, Hill et al. demonstrated depth-dependent HAXPES measurements of the two polarization states in LSMO/HfO_2_-based systems. The findings generally match well in showing spectral changes in the *P*_up_ state that resemble an increased vacancy concentration in the HZO and an oxidation of the LSMO, while the *P*_down_ state remains similar to the pristine state.^27^ Nevertheless, an in-depth comparison is inconclusive due to crucial differences in the experiment design. Namely, the different electrical biasing schemes via a piezoresponse force microscopy and the exposed oxide surface, leading to unscreened polarization charges and adsorbates from the environment that were shown to impact the core-level spectra significantly.

### Dual-Mode Operation

The identical STO/LSMO/HZO material system is common to two very different switching mechanisms that both originate from oxygen reorganization. What distinguishes them is the different biasing time scales and the spatial spread of the resulting chemical changes (cf. Figure 6c). With short biasing in the range of kHz pulses for ferroelectric switching, HAXPES revealed small chemical changes associated with vacancy migration across the whole device area. A DC bias instead comprises the regime of filamentary-type VCM switching. The oxygen-rich HZO here does not allow bulk vacancy movement or interface switching on DC time scales, as seen, for example, in La_0.67_Sr_0.33_MnO_3_/r-HZO or La_0.67_Sr_0.33_MnO_3_/BaTiO_3_/Pt.^25,61^ Therefore, accelerated by Joule heating,^54^ a distinct localized path of oxygen vacancies forms along structural variations, such as defects or grain boundaries, instead,^48,62–64^ clearly exceeding the suboxide emergence in the ferroelectric fatigue state. The penetration depth of the XPEEM measurements does not allow access to the underlying bottom electrode to determine the extent of chemical changes in the LSMO at the filament location. Nevertheless, we strongly suspect an LSMO contribution to the filament in the HZO, as illustrated in Figure 6c. Enabled by the stronger driving forces, such as DC bias and the corresponding temperature development, we propose a greater local involvement of LSMO compared to that observed in the ferroelectric regime. The semilocalized structures that show a slightly elevated vacancy concentration, Figures 2b and 3e, together with the LSMO contribution, can be assumed responsible for the distinct dual-mode operation unique to this material system. The facilitated filament formation without causing an irreversible soft breakdown allows the system to restore its pristine resistance after RESET, as suggested in prior work on the switching characteristics of the system.^8^

## Conclusions

Oxygen vacancy dynamics were observable across ferroelectric and resistive switching. In the case of the valence change mechanism-based filamentary switching, a pronounced and highly localized reduction occurred within the filament region. In contrast, areas beyond the filament displayed weaker, more distributed reduction throughout the device area. In contrast, in the ferroelectric switching mode, field cycling leads to an overall reduction in the HZO layer, with oxygen vacancies preferentially accumulating at the LSMO/HZO (bottom) interface. Notably, the vacancy distribution allows differentiation between the two polarization directions. The observations suggest that the LSMO contributes to oxygen exchange by alternately incorporating and releasing oxygen corresponding to the device’s state. However, the majority of redistribution and the newly generated vacancies remain inhomogeneously distributed in the HZO. The involvement of the LSMO bottom electrode and inherent preferential paths of oxygen vacancy accumulation in HZO enable the unique dual-mode operation of the LSMO/HZO system. This behavior underscores the critical role of the interface and bottom electrode in facilitating oxygen vacancy redistribution. An improved vacancy transport into the LSMO and reduced pinning at the interface might significantly reduce the ferroelectric degradation and could potentially further improve the resistive switching reliability, too.

## Experimental Section

### XPEEM Experiments

Twenty nm La_0.8_Sr_0.2_MnO_3−δ_ were grown on SrTiO_3_ substrates by pulsed laser deposition at 850 °C, 0.24 mbar oxygen pressure, 1.5 J cm^–2^ laser fluence. Eight nm Hf_0.5_Zr_0.5_O_2−δ_ were grown onto the STO/LSMO by pulsed laser deposition at the same temperature and laser fluence and at 0.1 mbar oxygen pressure. The sample was cooled down in 200 mbar oxygen atmosphere at 10 °C min^–1^. Further details on the fabrication and electrical characteristics can be found in.^8^ Devices were created by etching the HZO and LSMO by a positive photolithography process and ion beam etching. The etched thickness was filled up with an insulating Ta_2_O_5_ layer by sputter deposition. A monolayer of graphene was deposited on the sample via wet transfer and patterned into elongated rectangles perpendicular to the device orientation by photolithography and oxygen plasma etching. The resulting device area is the cross-section of the graphene strip and the LSMO/HZO fin. Lastly, Pt/Au contact leads by evaporation and lift-off, contact the graphene on both sides outside the device area. The devices were electrically connected to the sample holder by Al wire bonding. All resistive switching was performed using a Keithley 2611A SourceMeter, with the voltage applied to the top electrode. The XPEEM experiments in Figure 2 were performed at the MAXPEEM beamline at MAX IV synchrotron Lund, Sweden, and Figure S4 at the Nanospectroscopy beamline at Elettra synchrotron laboratory, Trieste, Italy. Image stacks were acquired at a photon energy of 200 eV at increasing kinetic energies with a step size of 100 meV for core-level spectra. Reference spectra were extracted from regions close to the filament. Spectra were extracted from the image stacks using the IGOR Pro software. A Shirley background and Voigt profiles were used to model the Hf 4*f* doublets in Figure 3a using KolXPD. The branching ratio was fixed according to the spin–orbit splitting, and the peak separation was fixed to 1.71 eV. The peak position was fixed for the fitting of the filament spectrum, as the broadening justifies an additional doublet. The position was not fixed for the spotty area to account for the rigid shift of the spectrum. The peak shape was kept constant for all fits.

### Conductive Atomic Force Microscopy

The C-AFM is performed with a Scienta Omicron VT-SPM at room temperature under a base pressure below 10^–9^ mbar. The measurements are performed with a sample bias, applied with a Keithley 2401, while the tip is grounded to the Omicron Matrix controller. Conductive single crystal diamond probes, Bruker AD-2.8-SS, with tip radius <5 nm were used. The force set point between the cantilever and the sample surface is 1.3 nN, and the scan speed is about 167 nm s^–1^.

### HAXPES Experiments

LSMO and HZO were grown on STO via pulsed laser deposition, as described before. A 3 nm Pt layer was evaporated. Rectangular devices of 190 × 50 μm^2^ were created by photolithography and ion beam etching of the Pt and HZO layer. An insulating Ta_2_O_5_ layer with a small overlap onto the device was deposited by sputter deposition and a lift-off process was used to open the device areas. Conductive leads to the devices were created by Pt/Au evaporation and a lift-off process. An electrical connection to the sample holder was established by Au wire bonding at the sample edge and Pt paste. Ferroelectric switching was performed with an aixACCT TFAnalyzer3000, with the voltage applied to the top electrode. Figure S7 provides a comparison of the in situ and ex-situ current response following the PUND scheme. The HAXPES measurements were performed at the GALAXIES beamline at the synchrotron radiation facility SOLEIL, France. In all cases, a photon energy of 6 keV with photon bandwidth 0.8 and 200 eV pass energy of the analyzer were used, horizontally polarized, colinear with the analyzer lens axis. The total resolution was 0.814 eV. All measurements were performed at room temperature. The takeoff angle was varied between 20, 45 and 60°, relative to the surface of the sample, to obtain a qualitative difference between the top and bottom interface of the HZO. The devices were switched in situ and remained grounded throughout measurements. The Pt 4*f*_7/2_ peak was fitted with a Donjach–Sunjich peak shape and referenced to 71 eV to align the binding energy of all spectra. A Shirley background and Voigt profile were used to model the Hf 3*d*_5/2_ peak using KolXPD. The peak shape was kept constant for all fits of the same takeoff angle, including the suboxide peak. The energy splitting between full oxide and suboxide peak was kept constant at 0.83 eV for all angles.

## Supplementary Material



## Notes

XRD measurement of STO/LSMO/HZO, AFM scan of HZO (Figure S1); resistive switching curves in different conditions (Figure S2); oxygen vacancy density calculation (Equation S3); XPEEM filament image in alternative device structure, XPEEM spectra of alternative device structure (Figure S4); XPEEM Hf 4f measurements of two devices, measured in the pristine state (Figure S5); HAXPES Hf 3*d* spectra at 45 and 60° takeoff angle (Figure S6); PUND measurement in- and ex-situ for positive, negative voltage (Figure S7); HAXPES Mn 2*p* difference spectra (Figure S8); HAXPES Mn 2*p* spectra: initial and after continuous hard X-ray exposure (Figure S9) (PDF)

## ○

Next-Generation Fuel Cell Research Center, Kyushu University, 744 Motooka, Nishi-ku, Fukuoka, 819–0395, Japan

## ¶

Department of Materials Science and Engineering, Massachusetts Institute of Technology, Cambridge, Massachusetts 02139, United States.
