# Supplementary material for: Oxygen Vacancy Dynamics in Different Switching Modes of Hf0.5Zr0.5O2−δ
Source: ACS Nano. 2025 Aug 6;19(32):29405–15. doi: 10.1021/acsnano.5c07038 (PMC12368994; doi:10.1021/acsnano.5c07038)
Supplement: Supplementary file 1 [file nn5c07038_si_001.pdf]

Supporting Information

Oxygen Vacancy Dynamics in Different Switching Modes of $Hf_{0.5}Zr_{0.5}O_{2-\delta}$

Judith Knabe, Kalle Goss, Yen-Po Liu, Evangelos Golias, Alexei Zakharov, Iulia Cojocariu, Matteo Jugovac, Andrea Locatelli, Tevfik O. Menteş, Denis Céolin, Alexander Gutsche, Daisy Gogoi, Moritz L. Weber, Rainer Timm, Regina Dittmann**

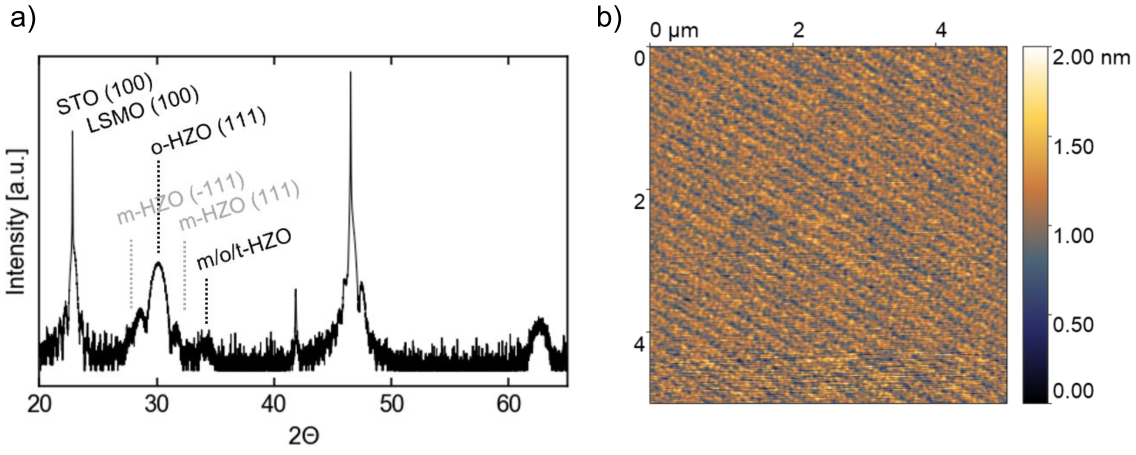

***Figure S1**: a) XRD measurement of a STO/LSMO/HZO stack, representative of all samples used for XPEEM and HAXPES investigations. b) AFM scan of the XPEEM device in Figure 2a in the main manuscript, before the graphene top electrode was transferred.*

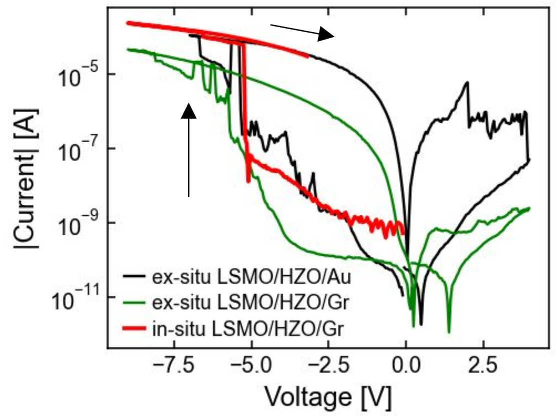

***Figure S2:** Comparison of resistive switching in different setups and with different top electrodes. Devices with graphene top electrode are in the XPEEM experiment geometry (see manuscript). The in-situ switching corresponds to the device in Figure 2b into the low resistive state prior to XPEEM measurement. Different current levels in the low resistive state can be assigned to the different setups used for biasing. All samples have an approximate HZO thickness of 8nm.*

Equation S3: Calculation of the oxygen vacancy density in the filament $N_{VO}[\frac{1}{m^3}]$. With the densities ρ, molar masses M and Avogadro constant N_A:

$$N_O = \frac{\frac{\rho_{HfO_2}+\rho_{ZrO_2}}{2}}{\frac{(M_{Hf}+M_{Zr})}{2}+2M_O} N_A * 2 \quad ; \quad N_{VO} = N_O * \frac{\#VO[\%]}{100}$$

Equation S3: *Calculation of the oxygen vacancy density in the filament, given a vacancy concentration.*

Figure S4: XPEEM investigations were further extended to simpler device architectures, to underline the representability of the findings utilizing graphene top electrodes. In Figure S4, devices of STO/LSMO/HZO/Au were prepared and switched into the low resistive state ex-situ. The Au top electrodes were consequently delaminated in-situ, preventing the filament re-oxidation and allowing access to the bare oxide surface for XPEEM measurements. The Hf4*f* measurement in Figure S4a shows a circular feature with lower intensity at higher binding energies, compared to the surrounding. Figure S4b shows the extracted spectra of the feature-core and a reference, showing a clear suboxide contribution comparable to the findings in Figure 3a in the main manuscript. Here, the fittings were done using CasaXPS and GL(30) doublets with a Shirley background. The measurement was conducted at the Nanospectroscopy beamline at Elettra synchrotron laboratory, Trieste, Italy.

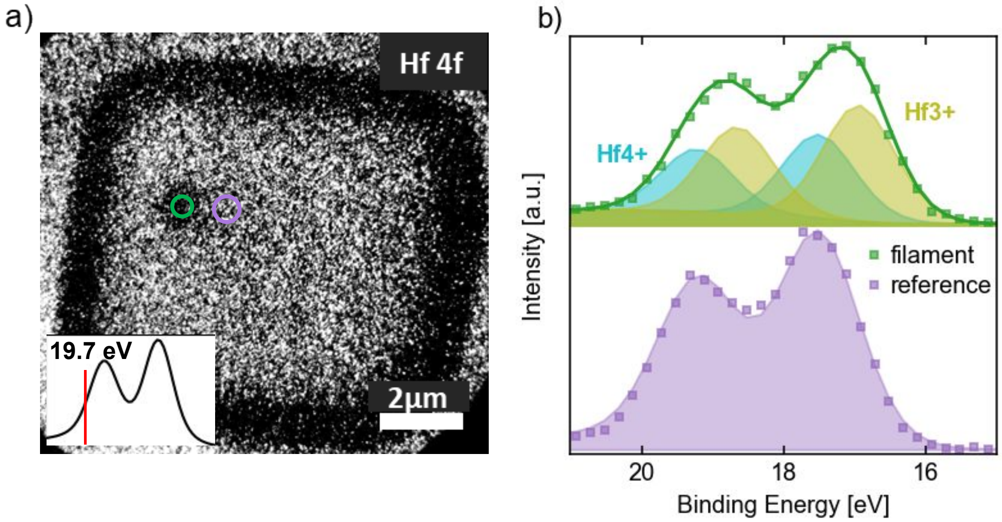

Figure S4: *More conventional (graphene free) device structure with in-situ delaminated Au top electrode. a) XPEEM Hf4f image taken at 19.7eV. b) XPEEM Hf4f measurement (squares) and fits (line) reveal a similar suboxide part (green, 14.5%) and filament diameter (100nm) as in Figure 1a and Figure 2 in the main manuscript.*

Figure S5: XPEEM Hf4*f* measurements of two further devices, measured in the pristine state, show no spotty structures as observed in the LRS in Figure 2b in the main manuscript. The images are extracted at the same relative positions in the Hf4*f* spectrum as in Figure 2b.

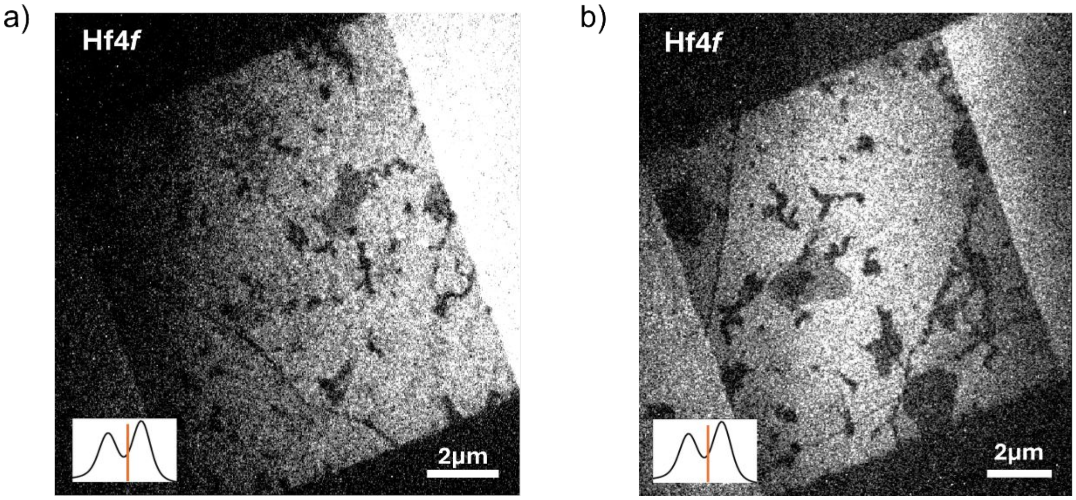

***Figure S5**: XPEEM* Hf4*f measurements of additional, equivalent devices. Both, a) and b) in the pristine state showing no signs of the spotty structures found in Figure 2b. The sketched spectrum in the bottom left indicates that the image is extracted at a similar relative position as in the main manuscript.*

Figure S6: Operando HAXPES measurements were performed at three distinct detection angles and in four different states. In addition to the spectra in Figure 4e of the manuscript, which was acquired at a take-off angle of 20° (most surface-sensitive angle), Hf $3d_{5/2}$ spectra obtained at 45° and 60° are displayed here, along with their corresponding peak fits. The Pt $4f_{7/2}$served as a reference and a Shirley background and Voigt profile were used to model the Hf $3d_{5/2}$peak. The peak positions summarized in Figure 5 of the manuscript also incorporate the data presented here.

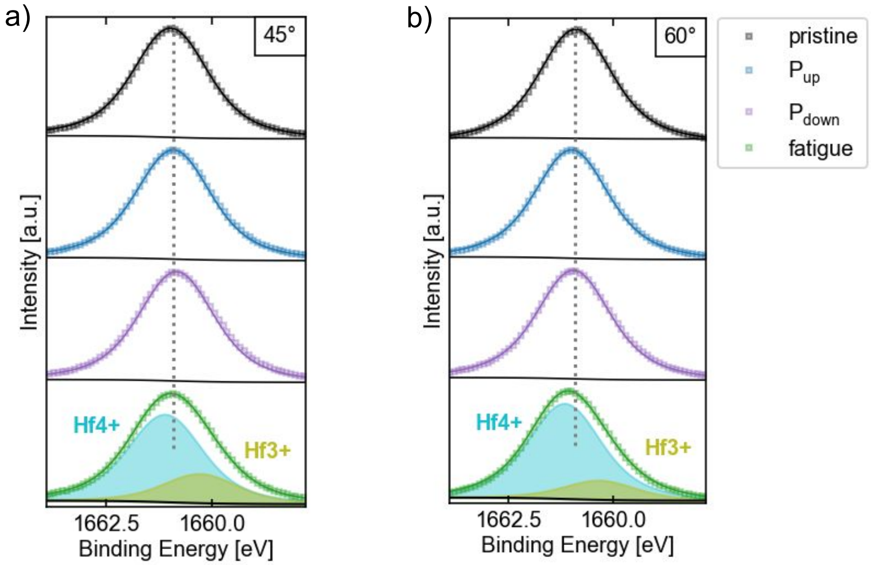

***Figure S6:** HAXPES measurements (squares) and peak-fits (line) of the Hf3$d_{5/2}$peak for the different states at a take-off angle of 45° and 60°. The data for 20° is shown in Figure 4e in the main manuscript.*

Figure S7: In-situ and ex-situ current response following the PUND-scheme of consecutive voltage pulses, using a ferroelectric tester (TFAnalyzer3000, aixACCT). The observed differences between in-situ and ex-situ switching behavior can primarily be attributed to the extensive additional connections required for in-situ addressing devices in the HAXPES chamber. The connection, via wire bonding, the sample holder and through the chamber to the external ferroelectric tester, significantly influences the high-frequency I-V response of ferroelectric switching, also justifying the higher voltages applied. Despite less pronounced, the presence of ferroelectric switching can still be identified by the characteristic difference between switching and non-switching pulses, here at $0.5{\cdot}10^{-4}$s.

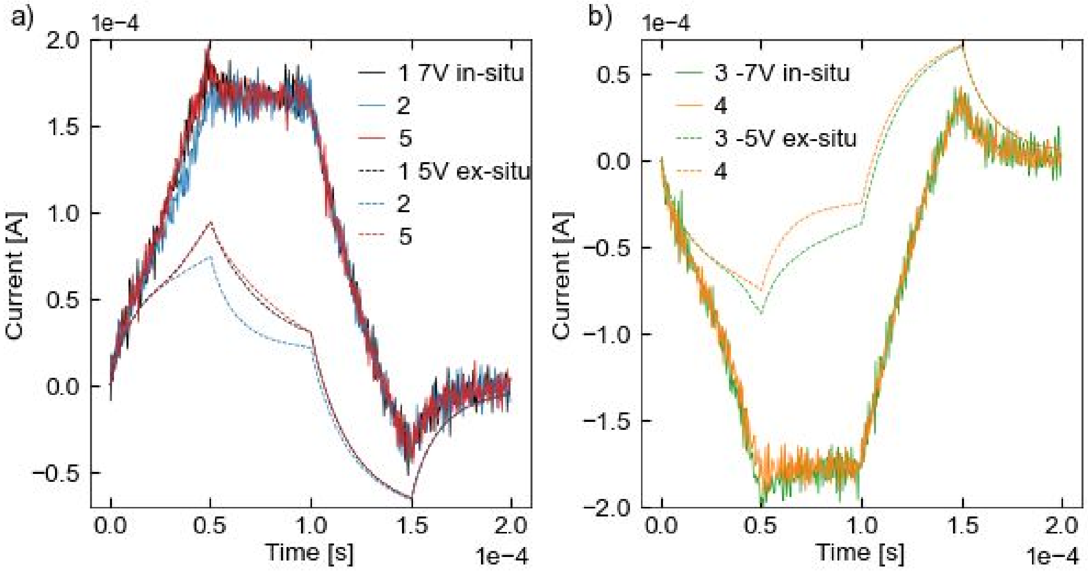

__Figure S7:__ PUND measurements of STO/LSMO/HZO/Pt devices used for HAXPES experiments. a) Comparison of the switching (1 & 5) and non-switching (2) current response in-situ and ex-situ, at +7V and +5V applied, respectively. b) Comparison of the switching (3) and non-switching (4) current response in- and ex-situ, at -7V and -5V applied.

Figure S8: The Mn2p spectra in Figure 6a,b in the main manuscript are here shown as difference-spectra. All spectra are normalized to the maximum and a linear background is subtracted. The pristine state spectrum is shown directly, all other spectra are shown with the pristine spectrum subtracted to highlight differences. At 45° all spectra show deviations consistent across the $Mn2p_{1/2}$ and $Mn2p_{3/2}$ peak. Considering the signal-to-noise ratio, a clearly higher intensity at higher binding energies and therefore, higher relative Mn4+ than in the pristine state is visible for the P_{up} state and to a smaller extent also for the P_{down} state. At 60° the differences to the pristine state are less coherent across the spin-orbit splitting. Nevertheless, an intensity reduction at lower binding energy (relative reduction of Mn3+) seems likely.

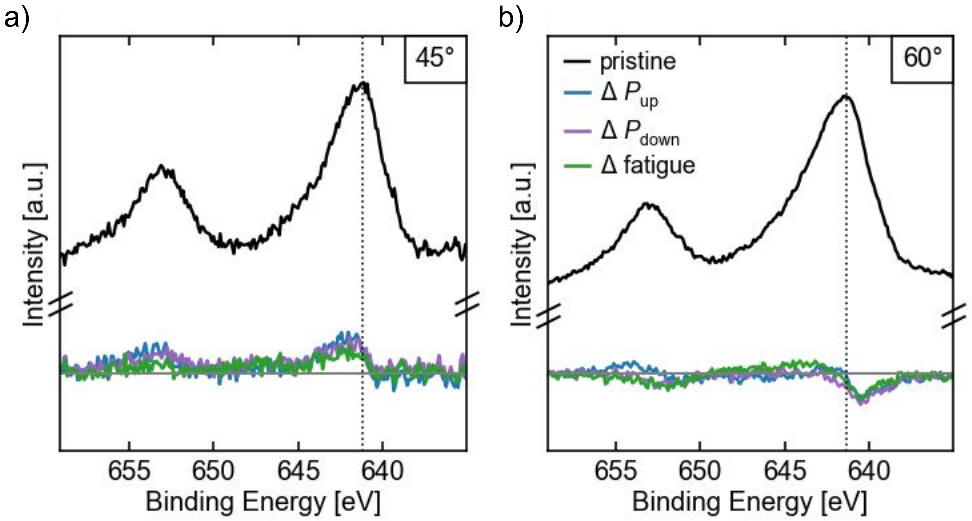

***Figure S8**: HAXPES Mn2p spectra measured in four different states. The pristine measurement is subtracted from the other states' spectra to highlight changes at a) ϴ=45° and b) ϴ=60°. The dashed line indicates the pristine peak maximum as a guide to the eye.*

Figure S9: Repeated Mn2*p* spectra are acquired to validate that the HAXPES measurement process itself does not induce any radiation-related changes in the sample. Figure S9 presents a comparison between the initial Mn2*p* spectrum and a subsequent spectrum acquired after a continuous exposure for 1 hour and 40 minutes. Both spectra were normalized, and a linear background subtraction was applied.
No significant variations are observed, particularly no indications of spectral shifts or peak asymmetry that would suggest a chemical reduction due to radiation-induced damage. These findings confirm the stability of the sample under the applied HAXPES measurement conditions.

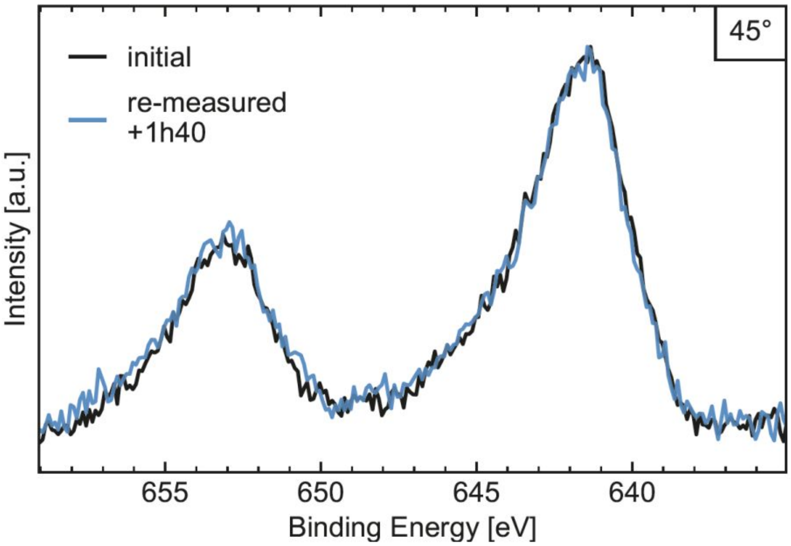

***Figure S9**: Mn2*p *core-level spectrum. The comparison of the initial spectrum with the repeated measurement after 1h40min demonstrates no alternation of the core-level spectra due to the hard X-rays. Spectra were normalized, and a linear background was subtracted.*
